# EEG signals respond differently to idea generation, idea evolution and evaluation in a loosely controlled creativity experiment

**DOI:** 10.1038/s41598-021-81655-0

**Published:** 2021-01-22

**Authors:** Wenjun Jia, Yong Zeng

**Affiliations:** Concordia Institute for Information Systems Engineering, Gina Cody School of Engineering and Computer Science, Concordia University, Montreal, QC Canada

**Keywords:** Cognitive control, Computational neuroscience

## Abstract

Many neurocognitive studies endeavor to understand neural mechanisms of basic creative activities in strictly controlled experiments. However, little evidence is available regarding the neural mechanisms of interactions between basic activities underlying creativity in such experiments. Moreover, strictly controlled experiments might limit flexibility/freedom needed for creative exploration. Thus, this study investigated the whole-brain neuronal networks’ interactions between three modes of thinking: idea generation, idea evolution, and evaluation in a loosely controlled creativity experiment. The loosely controlled creativity experiment will provide a degree of flexibility/freedom for participants to incubate creative ideas through extending response time from a few seconds to 3 min. In the experiment, participants accomplished a modified figural Torrance Test of Creative Thinking (TTCT-F) while their EEG signals were recorded. During idea generation, a participant was instructed to complete a sketch that was immediately triggered by a sketch stimulus at first sight. During idea evolution, a participant was instructed to complete a sketch that is radically distinctive from what was immediately triggered by the sketch stimulus. During the evaluation, a participant was instructed to evaluate difficulties of thinking and drawing during idea generation and evolution. It is expected that participants would use their experience to intuitively complete a sketch during idea generation while they could use more divergent and imaginative thinking to complete a possible creative sketch during idea evolution. Such an experimental design is named as a loosely controlled creativity experiment, which offers an approach to studying creativity in an ecologically valid manner. The validity of the loosely controlled creativity experiment could be verified through comparing its findings on phenomena that have been effectively studied by validated experimental research. It was found from our experiment that alpha power decreased significantly from rest to the three modes of thinking. These findings are consistent with that from visual creativity research based on event-related (de)synchronization (ERD/ERS) and task-related power changes (TRP). Specifically, in the lower alpha band (8–10 Hz), the decreases of alpha power were significantly lower over almost the entire scalp during idea evolution compared to the other modes of thinking. This finding indicated that idea evolution requires less general attention demands than the other two modes of thinking since the lower alpha ERD has been reported as being more likely to reflect general task demands such as attentional processes. In the upper alpha band (10–12 Hz), the decreases of alpha power were significantly higher over central sites during the evaluation compared to idea evolution. This finding indicated that evaluation involves more task-specific demands since the upper alpha ERD has been found as being more likely to reflect task-specific demands such as memory and intelligence, as was defined in the literature. In addition, new findings were obtained since the loosely controlled creativity experiment could activate multiple brain networks to accomplish the tasks involving the three modes of thinking. EEG microstate analysis was used to structure the unstructured EEG data to detect the activation of multiple brain networks. Combined EEG-fMRI and EEG source localization studies have indicated that EEG microstate classes are closely associated with the resting-state network as identified using fMRI. It was found that the default mode network was more active during idea evolution compared to the other two modes of thinking, while the cognitive control network was more active during the evaluation compared to the other two modes of thinking. This finding indicated that idea evolution might be more associated with unconscious and internal directed attention processes. Taken together, the loosely controlled creativity experiment with the support of EEG microstate analysis appears to offer an effective approach to investigating the real-world complex creativity activity.

## Introduction

### Conceptual models of creativity and empirical findings

Creativity is defined as the ability to generate novel, useful, and surprising ideas^1,2^. A large number of studies endeavour to develop conceptual models of creativity, which is believed to be non-deterministic, ill-structured, and unpredictable. Divergent and convergent thinking processes have been credited to two fundamental processes underlying creativity^3^. This dual-process model has evolved into a ‘Geneplore’ model that is constituted by generative and exploratory processes^4^. In the generative phase, one constructs an initial idea. In the exploratory phase, the initial idea is interpreted, modified, and regenerated, leading to creative products. The exploratory process can be viewed as interactions among many sub-processes underlying creativity such as idea interpretation, idea generation, idea evaluation, and overcoming fixation. An integrated dual process model of creativity has been proposed to systematically describe shifting between idea generation and idea evaluation^5^. In the field of design, a dual process model has been proposed to model the design process, which includes associative and inference engines^6^. The associative engine facilitates divergent transformations while the inference engine facilitates convergent transformations. In addition to the dual process models of creativity, a nonlinear evolution model has been proposed to represent design creativity^7,8^. The model indicates that design reasoning is a nonlinear process during which design problems, design solutions, and design knowledge all evolve simultaneously to highly unpredictable solutions that might be creative. Similarly, a creative design process has been modelled as a co-evolutionary process between the design problem and the design solution spaces^9–11^. Indeed, the fundamental design process is a reasoning process following recursive logic^12^. Along the same line, the Dynamic Universal Creativity Process (DUCP) captures dynamic characteristics of creativity in time and space through four mechanisms: continued exploration, concatenation, estimation, and exaptation^13^. Generally, the creative process is a unique and universal one that applies all kinds of activities across different domains that make use of creative mind-sets, analytical thinking, judicial thinking, and synthetic thinking^14^.

Accordingly, to understand the complex phenomena involved in creativity, it is necessary to investigate the following:

- each and all basic activities characterizing the complex phenomena of creativity;
- interactions among the basic activities and how they form the complex phenomena comprising creativity.

Similarly, Fink and Benedek (2014) identified three main challenges for creativity research^15^: (1) Conceptual models of creativity need to be clarified to distinguish creativity from classic mental abilities and to decompose the construct of creativity into definable neurocognitive processes; (2) Integration of structural and functional methods need to be developed to identify the definable neurocognitive processes during creative thinking; and (3) Studying more complex and real-life creativity.

Recent neurocognitive research aims to understand the neurophysiological mechanism of the basic activities underlying creativity, such as creative idea generation and idea evaluation. Creative idea generation and idea evaluation are two critical, basic activities in the dual process creativity models. Creative idea generation refers to generating novel and useful ideas while idea evaluation refers to selection of ideas^16,17^. EEG-studies have indicated that the creative idea generation is associated with increased alpha power, which is particularly significant at prefrontal and right posterior parietal sites^18,19^. Similarly, more strongly increased alpha power over right fronto-temporal sites was observed during humour comprehension tasks compared to during alternate uses tasks^20^. This finding support the argument that humour comprehension and creative design processes are twins^21^. When creative idea generation receives interference from fixation effects, alpha power increases at frontal sites throughout the period preceding the ideas^22^. The time-course of creative idea generation has been characterized as a U-shaped function of alpha power changes^23–26^. These alpha power changes over different cortical sites can be used to predict idea originality, as shown in a study of serial order effects during divergent thinking production^27^. In an effort to test whether designers’ stresses and mental effort exhibit an inverse U-shaped relationship in real-life creativity acts, it was found that alpha power significantly increased at low and medium levels of stress compared to high levels of stress during conceptual design^28^. However, changes in alpha power were not significantly different between the first and second halves of the design process. Distinct from findings in the time-course of creative idea generation^23,26^, this inconsistency might result from the experimental design in which the design process^28^ was less controlled. Idea evaluation has been associated with increases in alpha power, which has positive effects on the originality of idea generation^29^. These findings suggest that increases in alpha power are associated with inhibitory task-irrelevant stimuli and heightened internal attention^15^.

Regarding the basic activities of creativity in the figural domain, creative idea generation has been associated with decreased alpha power at parietal and occipital sites^30,31^. A few fMRI studies have indicated that creative idea generation is associated with activation of media temporal lobe and cerebral–cerebellar interactions, while idea evaluation is associated with activation of the executive network, default mode network, rostrolateral prefrontal cortex, insula, and temporopolar cortex^32,33^. In addition, in an effort to study creative idea generation, Rominger et al. (2018) considered two subprocesses of idea generation and idea elaboration, during which the latter elaborates a previously generated idea^30^. It was found that the decreases in alpha power were significantly reduced during idea elaboration compared to idea generation. Follow-up research indicated increases in functional coupling from idea generation to idea elaboration, which were significant in frontal-central and frontal-temporal networks^34^. In line with these findings, the significant power decreases in the lower alpha band were associated with figural divergent tasks compared to verbal divergent tasks, whereas the significant differences were observed only over occipital and left frontal sites between figural and verbal divergent tasks in the upper alpha band^35^. These findings suggest that brain responses are significantly different in creativity between the verbal and figural domains.

In summary, existing neurocognitive studies have made considerable progresses in understanding the basic cognitive activities underlying the complex phenomena of real life creativity, such as ideal generation, evaluation, and elaboration, through strictly controlled creativity experiments. While those progresses laid a solid foundation for creativity research, the interactions among basic creativity activities remain a largely open research area since they occur only in a more ecologically valid experimental setting.

### Issues with research methodology

A strictly controlled creativity experiment involves observing the effects of a creativity activity on brain responses with control of all extraneous variables^36^. Such an experimental paradigm provides a reliable approach to identifying repeatable/reproducible causal relations between a stimulus and a response^36^. However, a strictly controlled creativity experiment cannot simulate the interactions among creativity activities since one cannot control whether, when, or how a participant generates creative ideas. Moreover, the strictly controlled creativity experiment ignores the effects of spontaneous processes on creative thinking. For instance, incubation and mind-wandering, which are less controlled processes, have positive effects on creative thinking^37–40^. Furthermore, strict control of response time, such as 15 s, might inhibit the search for originality^27^. Indeed, creativity is not a repeatable, reproducible, or controllable act in that the same person may not produce the same result or follow the same pathway when he/she solves the same problem at two different times; two different people may not arrive at the same result or follow the same processes when given the same problem^8^. Therefore, there is a conflict between the need to strictly control an experiment for repeatability/reproducibility in identifying causal relations and the demand for freedom and flexibility to trigger the occurrence of creative ideas.

The considerable progress in analysing mechanisms of creativity could be attributed to analysing EEG signals by characterizing temporal waveform morphology and frequency distribution of EEG signals at certain channels, such as event-related potentials (ERPs) analysis, spectral analysis, and coherence analysis^41^. However, these approaches have not taken full advantage of multi-channel EEG signals due to neglecting the multivariate characteristics of these measurements. For instance, ERD/ERS^42^ and TRP^43^ are the most common spectral analysis methods for measuring power differences in specific frequency bands under varying conditions. They still have not taken into account the spatial configuration of electric fields at the scalp, nor have they fully considered temporal dynamics within these conditions.

EEG microstate analysis is an alternative and promising method for characterizing the configuration of the scalp potential field across and within conditions (see Michel and Koenig (2018) for a comprehensive review^44^). EEG microstates in the brain are defined as semi-stable configurations of the scalp potential field during successive short time periods, suggesting quasi-simultaneity of activity in distributed and large-scale brain networks^44^. Each semi-stable successive time period can be viewed as a basic cognitive function. EEG microstates can be detected by clustering-based and factor-based algorithms, such as modified k-means^45^, atomize and agglomerate hierarchical clustering (AAHC)^46^, principal component analysis (PCA)^47^ and independent component analysis (ICA)^48^. The theoretical properties of EEG microstate sequences are invariant using different EEG microstate detection methods^49^. Combined EEG-fMRI and EEG source localization studies have indicated that EEG microstates are closely associated with the resting-state networks as identified using fMRI^50–52^. Recent EEG studies have successfully used EEG microstate analysis to investigate the temporal dynamics of functional brain networks during complex cognitive tasks^53–57^. The authors’ group has also used EEG microstate analysis to segment complex design activities^58^ and to quantify the effort, fatigue, and concentration during the conceptual design process^59^. Compared to conventional spectral analysis, as shown in Table 1, EEG microstate analysis is more capable of structuring the EEG data into a set of semi-stable segments in which EEG changes are associated with changes in functional brain networks. This advanced method provides a promising approach for investigating whole-brain neuronal networks during complex tasks.

**Table 1:** Method comparison between conventional power analysis and EEG microstate analysis.

	ERD/ERS and TRP	Coherence	EEG micrsotate analysis
Experiment protocol: time	Fixed to a few seconds	Fixed to a few seconds	Self-paced and up to a few minutes and theoretically as many minutes as the experiment can last
Feature domain	Frequency	Frequency	Spatiotemporal
Data	Single electrode	Two electrodes	All of electrodes
Alignment with other neuroimaging techniques	N/A	N/A	fMRI

In summary, the issues with research methodology result from the experimental design and analytic methods. In strictly controlled experiments, it is difficult to simulate interactions between basic creative activities that trigger essential features of creativity, such as incubation. ERPs-, ERD/ERS-, and TRP-based analytic methods are not capable of capturing changes in spatial configuration of scalp electric fields between and within conditions. EEG microstate analysis may provide an alternative solution to enable more ecologically valid creativity experiments by structuring and analysing unstructured data collected through such experiments.

### Objective of the present study

In this study, our focus was to investigate the interactions among the basic activities and how they interact to form the complex phenomena that comprise creativity. First, we designed a loosely controlled creativity experiment corresponding to three modes of thinking: idea generation, idea evolution, and evaluation. The loosely controlled creativity experiment involved the observation of effects of three modes of thinking on brain responses without control of certain extraneous variables. Second, we used EEG microstate analysis to structure the unstructured EEG data into pairs of stimulus and response to decompose the complex higher order cognitive functions into a set of basic functional whole-brain neuronal networks. Such a method makes it possible to capture the repeatable/reproducible causal relations underlying the unstructured data collected from a loosely controlled experiment. The loosely controlled creativity experiment with the support of EEG microstate analysis appears to resolve a conflict between the need to strictly control a creativity experiment for repeatability/reproducibility and the demand to provide the freedom and flexibility for creative ideas to be incubated.

Task completion time was self-paced and lasted up to 3 min. Imaging and drawing phases were integrated into a single phase, and erasing to modify a drawn sketch was allowed. During idea generation, participants were expected to use their experience to simultaneously and intuitively generate an idea, which may not invoke creative thinking in participants. During idea evolution, participants were expected to generate novel, useful, and surprising ideas that were radically different from the previous ideas. Idea evolution is a recursive process during which the goal, knowledge, and idea co-evolves through interactive applications of divergent and convergent thinking^8,12,60–63^. Idea evolution is distinct from idea elaboration^30^, in which participants are instructed to elaborate and refine an existing idea to form a part of the final solution. Idea generation and idea evolution can be viewed as two parallel approaches to dealing with a sketch stimulus, which are two independent methods to approaching a creative problem, whereas idea generation and idea elaboration are two steps of creative idea generation. Of note, evaluation was not intended to ask participants to assess the ideas generated from either idea generation or idea evolution; instead, participants were asked to evaluate the difficulties of those two processes. Our focus was on the mode of thinking rather than the content of the thinking process.

Under a loosely controlled setting, the evaluative processes might happen during idea evolution in that participants might evaluate whether a complete sketch is creative according to their internal criteria. In addition, incubation and mind-wandering might occur during idea evolution in that participants could reframe the same problem and generate ideas that could be entirely different from those that were previously generated. The ability to inhibit common and intuitive ideas is critical for generating creative ideas through overcoming fixation^64–67^. Thus, such a loosely controlled setting can trigger some critical activities and interactions among them to incubate the genesis of creative ideas. A less controlled process might induce broad associations during the creative generation activities, which have been studied through behavioural, neurostimulation, and pharmacological interventions in an ecologically valid manner^68,69^. This loosely controlled experimental paradigm provides a less controlled approach for investigating the real-life creativity.

Therefore, we presented a loosely controlled creativity experiment to investigate whole-brain neuronal network interactions between three modes of thinking with the support of EEG microstate analysis. We hypothesized that decreases in alpha power would occur from rest to the three modes of thinking. Specifically, we hypothesized that decreases in alpha power would be significantly lower during idea evolution compared to idea generation and evaluation. In addition, we hypothesized that microstate properties would be significantly different between rest and the three modes of thinking, as well as between idea generation, idea evolution, and evaluation.

## Results

### Behavioural results

Task completion time was 54.047 s (SE=4.441) for idea generation, 92.785 s (SE=5.192) for idea evolution, and 16.476 s (SE=1.089) for evaluation. To test whether task completion time among participants was significantly different for similar tasks, task completion time of each run of similar tasks was normalized. Repeated measures ANOVA indicated that task completion time among participants was significantly different for idea evolution ($F(2,54)=4.762, p=0.012, \eta _p^2=0.150$), but was not significantly different for idea generation ($F(2,54)=2.339, p=0.106, \eta _p^2=0.080$) or evaluation ($F(1.570,42.398)=1.286, p=0.285, \eta _p^2=0.045$). A post hoc paired t-test revealed that task completion time decreased significantly from the first run to the second run of idea evolution ($t(27)=2.518, p=0.018, 95\% C=[0.282,0.277]$), whereas task completion time increased significantly from the second run to the third run of idea evolution $(t(27)=-2.562, p=0.016, 95\%C=[-0.292,-0.032]).$ However, task completion time was not significantly different between the first and third runs of idea evolution $(t(27)=-0.185, p=0.855, 95\%C=[-0.117,0.980]).$

To test whether task completion time among participants was significantly different for different tasks, task completion time of all tasks was normalized and averaged within idea generation, idea evolution, and evaluation, respectively. Repeated measures ANOVA indicated that task completion time among participants was significantly different amid idea generation, idea evolution, and evaluation ($F(2,54)=69.554, p<0.001, \eta _p^2=0.720$). A post hoc paired t-test revealed that task completion time decreased significantly from idea evolution to idea generation ($t(27)=5.805, p<0.001,95\% C=[25.046,52.429]$) and evaluation ($t(27)=11.811, p<0.001,95\% C=[63.052,89.566]$), as well as from idea generation to evaluation ($t(27)=5.991, p<0.001,95\% C=[24.705,50.738]$).

### EEG results

#### EEG alpha power

In the lower alpha band (8–10 Hz), the $3 \times 5 \times 2$ repeated measures ANOVA revealed three significant main effects, including CONDITION ($F(1.291,34.860)=8.373, p=0.004, \eta _p^2=0.237$), AREA ($F(1.952,52.693)=40.390, p<0.001, \eta _p^2=0.599$) and HEMISPHERE ($F(1.000,27.000)=5.383, p=0.028, \eta _p^2=0.166$), as well as one significant interaction effect for CONDITION $\times$ AREA ($F(4.358, 117.658)=3.199, p=0.013, \eta _p^2=0.106$).

**Figure 1:**
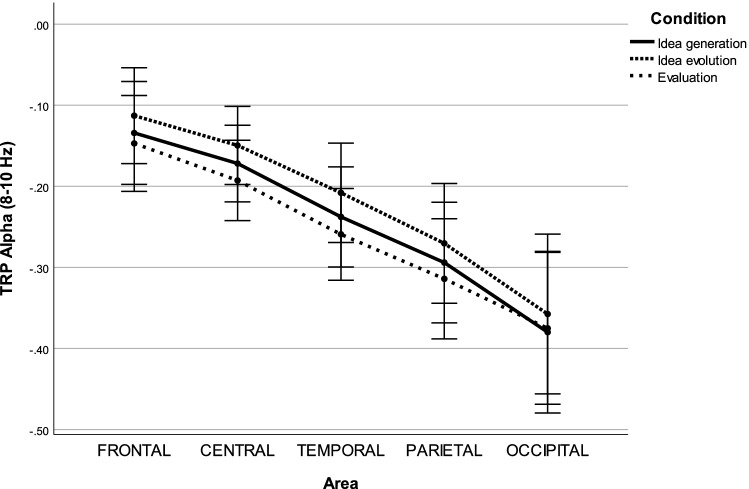
Task-related power in the lower alpha band during idea generation, idea evolution, and evaluation.

A post hoc test with Bonferroni correction on the main effect, CONDITION, indicated that decreases in alpha power were significantly lower during idea evolution compared to idea generation ($MD=0.024, p<0.001, 95\%C=[0.011,0.037]$) and evaluation ($MD=0.038, p=0.008, 95\%C=[0.009,0.067]$). For the main effect HEMISPHERE, decreases in alpha power were significantly lower over left hemispheric sites compared to right hemispheric sites ($MD=0.033, p=0.028, 95\%C=[0.004,0.062]$). For the main effect AREA, decreases in alpha power were significantly lower at frontal sites compared to temporal ($MD=0.104, p<0.001, 95\%C=[0.053,0.154]$), parietal ($MD=0.161, p<0.001, 95\%C=[0.089,0.234]$), and occipital sites ($MD=0.239, p<0.001, 95\%C=[0.149,0.330]$). Decreases in alpha power were significantly lower at central sites compared to temporal ($MD=0.064, p<0.001, 95\%C=[0.028,0.099]$), parietal ($MD=0.121, p<0.001, 95\%C=[0.060,0.182]$), and occipital sites ($MD=0.199, p<0.001,95\%C=[0.105,0.293]$). Decrease in alpha power were significantly lower at temporal sites compared to parietal ($MD=0.058, p=0.017, 95\%C=[0.007,0.109]$) and occipital sites ($MD=0.136, p<0.001, 95\%C=[0.069,0.203]$). Decreases in alpha power were significantly lower at parietal sites compared to occipital sites ($MD=0.078, p=0.002, 95\%C=[0.022,0.134]$). For the interaction effect CONDITION $\times$ AREA, decreases in alpha power were significantly lower during idea evolution compared to idea generation at frontal ($MD=0.021, p=0.017, 95\%C=[0.003,0.039]$), central ($MD=0.022, p=0.002, 95\%C=[0.008,0.037]$), temporal ($MD=0.030, p<0.001, 95\%C=[0.015,0.044]$), parietal ($MD=0.024, p=0.004, 95\%C=[0.007,0.040]$), and occipital ($MD=0.022, p=0.006, 95\%C=[0.006,0.039]$) sites. Decreased alpha power was significantly lower during idea evolution compared to evaluation at frontal ($MD=0.034, p=0.005, 95\%C=[0.009,0.059]$), central ($MD=0.043, p=0.010, 95\%C=[0.009,0.077]$), temporal ($MD=0.051, p=0.002, 95\%C=[0.018,0.084]$), and parietal ($MD=0.044, p=0.010, 95\%C=[0.009,0.079]$) sites (see Fig. 1).

In the upper alpha band (10–12 Hz), the $3 \times 5 \times 2$ repeated measures ANOVA uncovered one significant main effect, AREA ($F(1.782,48.111)=7.733, p=0.002, \eta _p^2=0.223$), as well as one significant interaction effect, CONDITION $\times$ AREA ($F(2.980,80.470)=4.139, p=0.009, \eta _p^2=0.133$).

**Figure 2:**
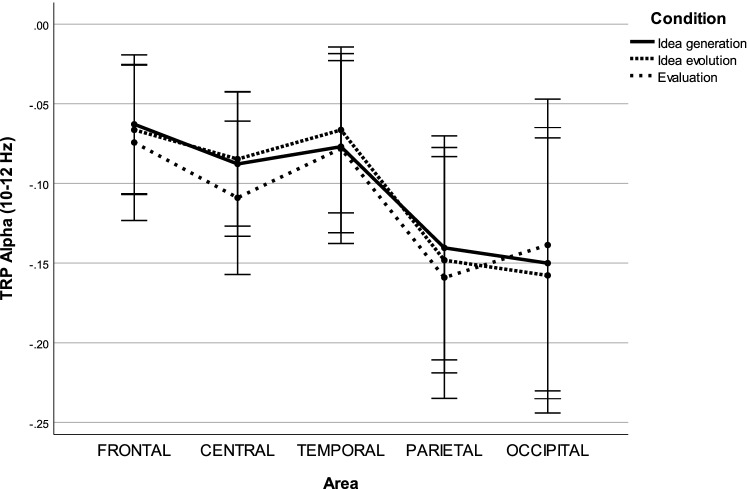
Task-related power in the upper alpha band during idea generation, idea evolution, and evaluation.

A post hoc test with Bonferroni correction on the main effect AREA indicated that decreases in alpha power were significantly higher at parietal sites compared to frontal ($MD=-0.081, p=0.021, 95\%C=[-0.154,-0.008]$), central ($MD=-0.055, p=0.040, 95\%C=[-0.109,-0.002]$), and temporal sites ($MD=-0.075, p<0.001, 95\%C=[-0.120,-0.031]$). In contrast, decreases in alpha power were significantly lower at temporal sites compared to occipital sites ($MD=0.075, p=0.012, 95\%C=[0.012,0.138]$). For the interaction effect CONDITION $\times$ AREA, decreases in alpha power were significantly lower during idea evolution compared to evaluation at central sites ($M=0.024, p=0.086, 95\%C=[0.001, 0.048]$) (see Fig. 2).

#### EEG microstate classes

According to cross validation, the optimal number of individual microstate classes was found to be 6.464 ($SE=0.166$) for rest, 6.250 ($SE=0.152$) for idea generation, 6.285 ($SE=0.168$) for idea evolution, and 6.857 ($SE=0.199$) for evaluation. Therefore, the number of individual microstate classes was defined as six for each run, each condition and each participant due to the comparability and simplicity of statistical analysis.

**Figure 3:**
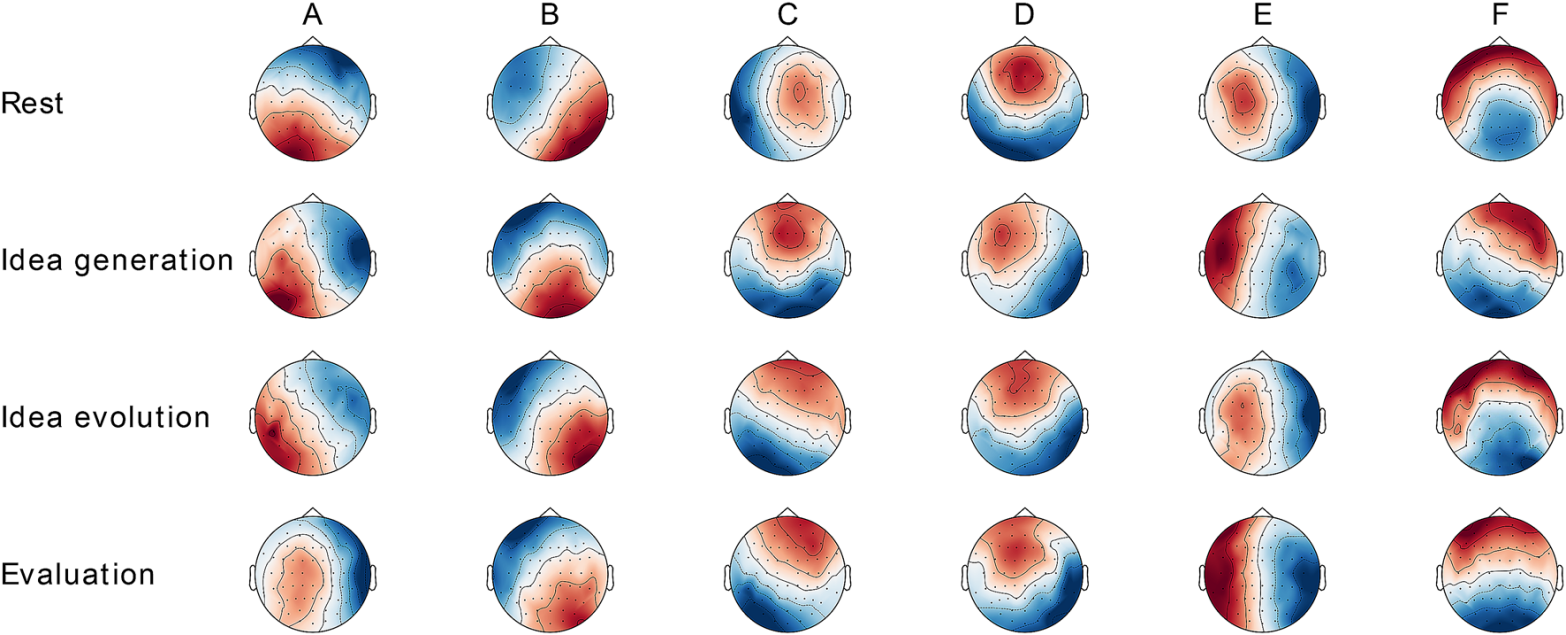
Condition-wise topographies of six microstate classes for the conditions of rest, idea generation, idea evolution, and evaluation. They are labelled and sorted according to Custo et al.^52^.

**Figure 4:**
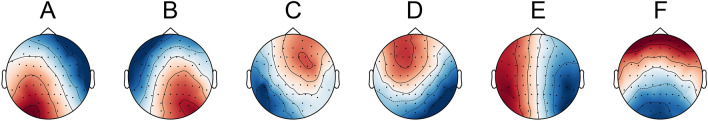
The topographies of six global microstate classes across runs, across participants, and across conditions. They are labelled and sorted according to Custo et al.^52^.

The six individual microstate classes explained 62.97% ($SE=1.111$) of the global variance of the original EEG topographies corresponding to peaks of GFP for rest, 60.87% ($SE=1.150$) for idea generation, 61.01% ($SE=1.108$) for idea evolution, and 61.46% ($SE=1.129$) for evaluation. Condition-wise EEG microstate classes across runs and participants are shown in Fig. 3. Global microstate classes across runs, across participants, and across conditions are shown in Fig. 4. The microstate classes A-F were labelled and sorted according to the literature^52^.

#### EEG microstate topographies

The $4 \times 6$ TANOVA revealed a significant main effect, CLASS ($p<0.001$). Neither the main effect CONDITION ($p=0.066$) nor the interaction effect CONDITION $\times$ CLASS ($p=0.804$) were significant.

Class-wise TANOVAs revealed a significant effect, CLASS C ($p=0.041$). Effects CLASS A ($p=0.838$), CLASS B ($p=0.610$), CLASS D ($p=0.740$), CLASS E ($p=0.383$) and CLASS F ($p=0.125$) were not significant.

**Table 2:** Paired TANOVA p-values for microstate topography between conditions.

Conditions			Microstate classes					
			Class A	Class B	Class C	Class D	Class E	Class F
Rest	Vs.	Idea generation	0.359	0.545	0.040*	0.707	0.137	0.153
Rest	Vs.	Idea evolution	0.257	0.317	0.041*	0.438	0.275	0.625
Rest	Vs.	Evaluation	0.136	0.381	0.025*	0.568	0.157	0.137
Idea generation	Vs.	Idea evolution	0.131	0.747	0.368	0.438	0.749	0.104
Idea generation	Vs.	Evaluation	0.733	0.476	0.879	0.800	0.946	0.305
Idea evolution	Vs.	Evaluation	0.014*	0.815	0.330	0.664	0.726	0.215

*$$\rho < 0.050$$,   **$$\rho < 0.010$$,    ***$$\rho < 0.005$$

Paired t-test revealed a significant effect CLASS C between rest and the three modes of thinking: idea generation ($p=0.040$), idea evolution ($p=0.041$) and evaluation ($p=0.025$), as well as a significant effect, CLASS A, between idea evolution and evaluation ($p=0.014$) (see Table 2).

#### EEG microstate parameters

For coverage of microstate classes, the $4 \times 6$ repeated measures ANOVA revealed a significant main effect, CLASS ($F(2.609,70.446)=19.214, p<0.001, \eta _p^2=0.416$), and a significant interaction effect, CONDITIONS $\times$ CLASS ($F(5.629,151.984)=7.081, p<0.001, \eta _p^2=0.208$).

**Figure 5:**
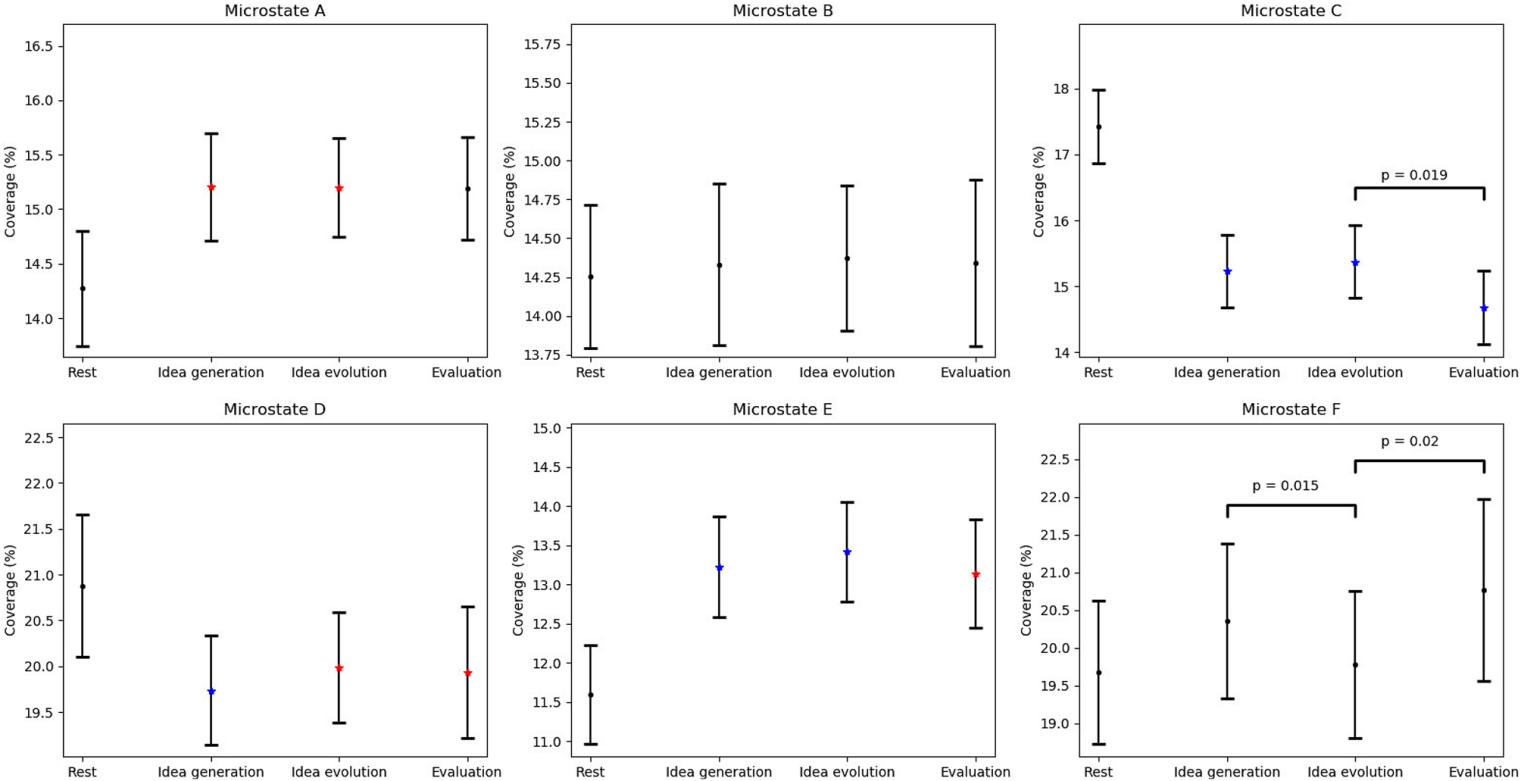
Error bar of microstate coverage for rest, idea generation, idea evolution, evaluation, and differences between the conditions. P-values are represented by the black dots ($\hbox {p}>0.05$), red asterisks ($\hbox {p}<0.05$), green asterisks ($\hbox {p}<0.01$), and blue asterisks ($\hbox {p}<0.005$) between rest and the three modes thinking. P-values are annotated between idea generation, idea evolution, and evaluation.

The paired t-test revealed a significant effect, CLASS A, between rest and idea generation ($t(27)=-2.522, p=0.017, 95\%C=[-0.017,-0.002]$), as well as idea evolution ($t(27)=-2.652, p=0.013, 95\%C=[-0.016,-0.002]$), indicating that coverage of microstate A was lower during rest compared to idea generation and idea evolution. The significant effect, CLASS C, indicated coverage of microstate C was higher during rest compared to idea generation ($t(27)=5.739, p<0.005, 95\%C=[0.014,0.030]$), idea evolution ($t(27)=5.017, p<0.005, 95\%C=[0.012,0.029]$), and evaluation ($t(27)=5.525, p<0.005, 95\%C=[0.017,0.038]$). The significant effect, CLASS D, indicated that coverage of microstate D was higher during rest compared to idea generation ($t(27)=3.368, p<0.005, 95\%C=[0.004,0.018]$), idea evolution ($t(27)=2.739, p<0.05, 95\%C=[0.002,0.016]$) and evaluation ($t(27)=2.637, p<0.05, 95\%C=[0.002,0.017]$). The significant effect, CLASS E, indicated that coverage of microstate E was lower during rest compared to idea generation ($t(27)=-3.460, p<0.005, 95\%C=[-0.026,-0.007]$), idea evolution ($t(27)=-3.845, p<0.005, 95\%C=[-0.028,-0.009]$) and evaluation ($t(27)=-2.745, p<0.05, 95\%C=[-0.027,-0.004]$). In Particular, coverage of microstate C was longer during idea evolution compared to evaluation ($t(27)=2.500, p=0.018, 95\%C=[0.001,0.013]$). Furthermore, coverage of microstate F was lower during idea evolution compared to idea generation ($t(27)=-2.594, p=0.015, 95\%C=[-0.010,-0.001]$) and evaluation ($t(27)=-2.483, p=0.019, 95\%C=[-0.018,-0.002]$) (See Table 3 and Fig. 5).

**Table 3:** Paired t-test p-values for microstate coverage between conditions.

Conditions			Microstate classes					
			Class A	Class B	Class C	Class D	Class E	Class F
Rest	Vs.	Idea generation	0.017*	0.853	0.001***	0.002***	0.001***	0.217
Rest	Vs.	Idea evolution	0.013*	0.722	0.001***	0.010*	0.001***	0.845
Rest	Vs.	Evaluation	0.056	0.815	0.001***	0.013**	0.010*	0.134
Idea generation	Vs.	Idea evolution	0.968	0.842	0.447	0.131	0.378	0.015*
Idea generation	Vs.	Evaluation	0.956	0.969	0.057	0.496	0.738	0.317
Idea evolution	Vs.	Evaluation	0.976	0.894	0.018*	0.862	0.277	0.019*

*$$\rho < 0.050$$,   **$$\rho < 0.010$$,    ***$$\rho < 0.005$$.

For duration of microstate classes, the $4 \times 6$ repeated measures ANOVA revealed a significant main effect CLASS ($F(2.443,65.949)=18.444, p<0.001, \eta _p^2=0.406$) and a significant interaction effect CONDITION $\times$ CLASS ($F(5.967,161.110)=4.439, p<0.001, \eta _p^2=0.141$).

**Figure 6:**
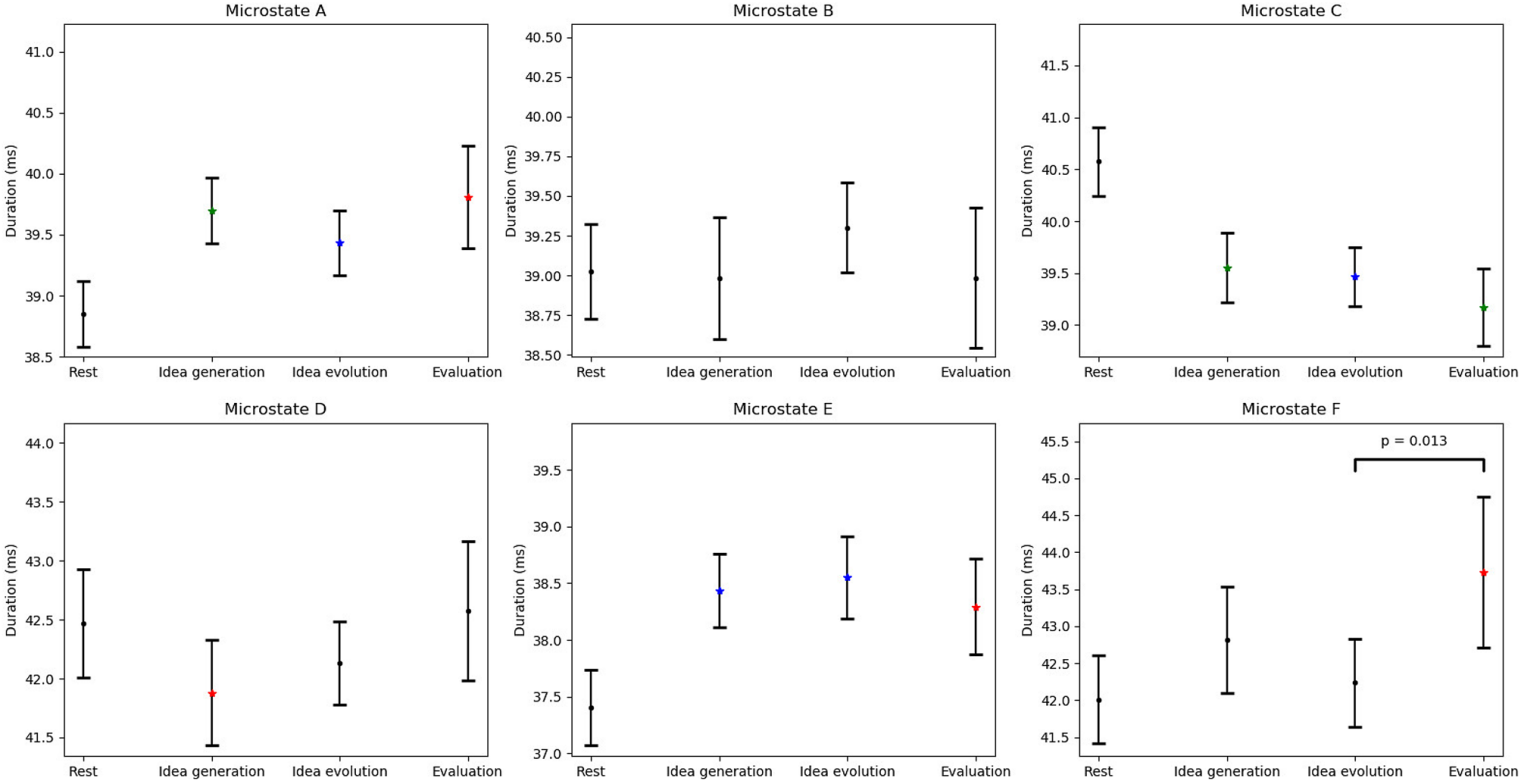
Error bar of microstate duration for rest, idea generation, idea evolution, evaluation, and differences between the conditions. P-values are represented by the black dots ($\hbox {p}>0.05$), red asterisks ($\hbox {p}<0.05$), green asterisks ($\hbox {p}<0.01$), and blue asterisks($\hbox {p}<0.005$) between rest and the three modes thinking. P-values are annotated between idea generation, idea evolution, and evaluation.

The paired t-test revealed a significant effect, CLASS A, between rest and idea generation ($t(27)=-3.032, p<0.01, 95\%C=[-0.708,-0.136]$), idea evolution ($t(27)=-3.131, p=0.004, 95\%C=[-0.481,-0.100]$), and evaluation ($(t27)=-2.175, p=0.038, 95\%C=[-0.930,-0.027]$), indicating that duration of microstate A was lower during rest compared to the three modes of thinking. The significant effect, CLASS C, indicated that duration of microstate C was higher during rest compared to idea generation ($t(27)=3.051, p<0.01, 95\%C=[0.167,0.852]$), idea evolution ($t(27)=3.561, p<0.005, 95\%C=[0.235,0.873]$), and evaluation ($t(27)=3.040, p<0.01, 95\%C=[0.228,1.173]$). The significant effect, CLASS D, indicated that duration of microstate D was higher during rest compared to idea generation ($t(27)=2.243, p=0.033, 95\%C=[0.025,0.563]$). The significant effect, CLASS E, indicated that duration of microstate E was lower during rest compared to idea generation ($t(27)=-3.686, p<0.005, 95\%C=[-0.805,-0.229]$), idea evolution ($t(27)=-3.529, p<0.005, 95\%C=[-0.909,-0.241]$), and evaluation ($t(27)=-2.172, p=0.038, 95\%C=[-0.867,-0.025]$). The significant effect, CLASS F, indicated that duration of microstate F was lower during rest compared to evaluation ($t(27)=-2.408, p=0.023, 95\%C=[-1.589,-0.127]$). In particular, duration of microstate F was lower during idea evolution compared to evaluation ($t(27)=-2.646, p=0.013, 95\%C=[-1.321,-0.167]$) (See Table 4 and Fig. 6).

**Table 4:** Paired t-test p-values for microstate duration between conditions.

Conditions			Microstate classes					
			Class A	Class B	Class C	Class D	Class E	Class F
Rest	Vs.	Idea generation	0.006**	0.899	0.006**	0.033*	0.001***	0.051
Rest	Vs.	Idea evolution	0.004***	0.249	0.002***	0.225	0.001***	0.486
Rest	Vs.	Evaluation	0.038*	0.904	0.006**	0.780	0.038*	0.023*
Idea generation	Vs.	Idea evolution	0.154	0.213	0.661	0.238	0.533	0.082
Idea generation	Vs.	Evaluation	0.705	0.995	0.296	0.051	0.628	0.158
Idea evolution	Vs.	Evaluation	0.279	0.303	0.394	0.210	0.377	0.013*

*$$\rho < 0.050$$,    **$$\rho < 0.010$$,    ***$$\rho < 0.005$$

## Discussion

Herein, we present a loosely controlled creativity experiment to uncover the interactions of whole-brain neuronal networks among three modes of thinking in an ecologically valid manner. This experiment provided sufficient flexibility and duration for participants to generate potentially creative ideas while maintaining certain levels of control by structuring the entire experiment into three sections, each of which was anticipated to simulate one of the three modes of thinking. First, we observed that alpha power decreased significantly from rest to the three modes of thinking. This finding is consistent with other more structured experimental studies in that alpha power decreased from rest to visual creativity^70^. Second, microstate topographies were significantly different between rest and the three modes of thinking, as well as between idea evolution and evaluation. Third, it was found that microstate parameters were significantly different between rest and the three modes of thinking, as well as between idea generation and idea evolution and between idea evolution and evaluation. The last two findings benefited from the loose controllability of the adopted experimental design.

### Why do we need a loosely controlled creativity experiment?

The behavioural results revealed that task completion time among participants was significantly different within idea evolution but was not significantly different within idea generation or within evaluation. This finding implies that the process of idea evolution is more flexible and unstructured than the processes of idea generation and evaluation. A post hoc paired t-test indicated that task completion time was significantly different between the first and second runs, as well as between the second and third runs of idea evolution. However, task completion time was not significantly different between the first and third runs of idea evolution. This finding implies that the same person may not follow a process in a similar way when generating solutions while solving similar creativity problems. In addition, a previous experiment on the co-evolutionary creative design process indicated that different people may not arrive at the same result or follow a process similarly when they solve the same creativity problem^10^.

Furthermore, task completion time for idea evolution was significantly higher compared to idea generation and evaluation. This result confirms that idea evolution may require more effort and implies more uncertainties due to its opened-ended goals and processes. Experiments reported by other researchers also confirm that a subject needs to spend more time defining and understanding a creativity problem to generate solutions recursively^10,71^.

Indeed, the process of creativity, which is believed to be unrepeatable, irreproducible, and uncontrollable, is co-evolutionary and follows recursive logic^9–12^. Recursive logic was first proposed by Zeng and Cheng (1991) to illustrate the nature of design thinking^12^. The logic was further formulated and formalized using set theory into a science-based design process model, which states that design problem, design knowledge, and design solutions evolve simultaneously in a design process^60,61^. Given a design problem, a designer will identify the relevant knowledge to generate a tentative design solution, which will improve the designer’s understanding of the design problem. This improved understanding might lead to a reformulation of the original design problem. The reformulated problem will lead the designer to identify new knowledge and to change the previous solution, which in turn leads to another reformulation of the design problem.

With a similar line of understanding, some scholars have noted the importance of recursion in the sub-phases of creativity^72,73^. In describing the design process, Gero and Kannengiesser (2004) highlighted that an “agent’s view of the world changes depending on what the agent does”^74^. In describing the creativity process, Corazza (2019) stressed that continued exploration is a primary force driving the recursion underlying the creativity process, while bidirectional dynamic interaction with the environment influences the recursion underlying the creativity process in terms of dynamic assessment and the emergence of unpredictable new functionalities^13^. Lubart (2001) indicated that initial ideas might interact with the developing work in a dynamic and evolving creative process^72^. Lubart also raised several key questions for future research on creativity, such as “to what extent is the creative processes recursive?”; “how exactly this recursion is organized?”; “what provokes recursion?”; and “what metacognitive functions control the choice of certain subprocesses and their recursive application?”. Nguyen and Zeng (2012) formulated the recursive design process into a nonlinear dynamic, for which a minor initial state difference may lead to huge state differences after many rounds of evolution^8^. Some of the new states can be credited as creative, whereas others can be identified as inconclusive outcomes that are not creative. The process of exploring the potentially original and effective ideas is still considered a creative process regardless of the originality or conclusiveness of the final outcome^75^. The nonlinear design dynamics imply a mechanism of creativity, which accommodates a degree of flexibility, uncertainty and unpredictability through a structured and deterministic model of design. Since a design problem often arises from a conflict in the current environment, which includes nature, human and artefacts, design knowledge is retrieved from a subset of relationships among environment components, and a design solution is always generated by synthesizing existing objects in the environment. Therefore, the nonlinear recursive design process can be naturally viewed as an environment-evolving process. The environment evolutionary process, termed Environment-Based Design (EBD), continues until the designer determines that a design solution is satisfactory^62^.

It is obvious from both the experimental and theoretical observations described above that flexibility/freedom is a fundamental need to incubate creativity through sufficient duration and open-ended tasks. The sufficient duration might induce a period of incubation and mind wondering that could facilitate creative problem solving through relaxation, overcoming fixation, and mental set-shifting^37–40^. Open-ended tasks offer unlimited potential for participants to explore solutions without predefined solutions or strategies. The ill-defined nature of this method, which is the most important characteristic of open-ended tasks, provides a degree of uncertainty of solutions in that an intermediate solution may redefine the original task from which new tasks may emerge. Different emergent tasks will induce different knowledge and solutions, which will, in turn, redefine the original tasks.

In order to achieve statistically significant results, conventional creativity tests, which are strictly controlled, limit participants’ flexibility/freedom. Dietrich (2019) observed that conventional creative tests do not have sufficient ecological validity due to the lack of considerations of multifaceted creativity^76^. Agnoli et al. (2020) also indicated that insufficient duration (15 s) might inhibit the search for originality such that a less constrained experimental setting is needed in future research^27^. Moreover, conventional creativity tests are focused on a single phenomenon of a creative process that includes idea generation, idea elaboration or idea evaluation. Zeng et al. (2011) indicated that the lack of understanding of interactions between the phenomena of a creative process is a major shortcoming of conventional creativity tests^73^.

Therefore, both behavioural findings and theoretical findings have suggested that creativity needs to be studied through a loosely controlled creativity experiment. The purpose of loosely controlled creativity experiments is to encourage participants’ natural characteristics of creativity in ways that can be analysed using a statistically reliable and significant approach. The loosely controlled creativity experiment concentrates on complex creativity activities, during each of which different creative phenomena are involved since it is believed that these phenomena are interdependent. Idea generation, idea evaluation, and idea elaboration, as suggested by Ellamil et al.^32^, Hao et al.^29^, and Rominger et al.^30^, might occur at a different time during idea evolution. Idea generation could be generally associated with a bottom-up and spontaneous process, whereas idea evaluation could be related to a top-down and controlled process. Idea elaboration cannot continue without a recursive implementation of idea generation and idea evaluation. Recently, complex creativity activities gradually attracted the attention of scholars in the field of design. For instance, Alexiou et al. (2009) designed an open-ended task that required not only solution generation but also problem understanding and solution evaluation^77^.

Nevertheless, loosely controlled creativity experiments increase the difficulty of data analysis since it triggers complex pairs of stimuli and response underlying the unstructured EEG data. Nguyen and Zeng (2014) conducted a preliminary analysis of the EEG spectrogram of a single subject from the loosely controlled creativity experiment, which is further analysed in the present paper^78^. The authors’ lab has been identifying the regularities underlying the unstructured data from the loosely controlled creative process. Nguyen et al. (2019) proposed microstate- and frequency-based methods to segment unstructured EEG data produced from complex design activities^58^. It was found that the best segmentation algorithm, which is the microstate-based method, has an average deviation of 2-s from manual segmentation. Each structured segment can be associated with a primitive design activity. Moreover, EEG microstate analysis has been used to structure unstructured data in the goal-free and goal-directed tasks by different authors^50–57^. Each microstate class could be associated with a specific whole-brain network, which has been effectively studied through fMRI. Accordingly, the loosely controlled creativity experiment with the support of EEG microstate analysis, known as a task-related EEG (tEEG) framework^79^, appears to offer an effective approach for studying real-world complex creativity activities. This study further refines and validates the tEEG framework by investigating the three modes of thinking in an ecologically valid manner.

### Is a loosely controlled creativity experiment valid?

The validity of the loosely controlled creativity experiment was verified by comparing its findings on phenomena that have been effectively studied by validated experimental research. It was found that alpha power decreased significantly from rest to the three modes of thinking. These findings are consistent with those from ERD/ERS- and TRP-based research on visual creativity (see Pidgeion et al. (2016) for a comprehensive review)^70^. Both of ERD/ERS and TRP revealed characteristics of neural oscillations in specific frequency bands among different conditions. These frequency features were independent of task completion time. That is, significant differences in task completion time did not affect the results that were analysed by the ERD/ERS or TRP. Therefore, it is reasonable and feasible to compare findings based on ERD/ERS and TRP to verify the validity of the loosely controlled creativity experiment.

It was found that alpha power changes were more sensitive during the three modes of thinking in the lower alpha frequency band (8–10 Hz) than that in the upper alpha frequency band (10–12 Hz), as shown in Fig. 1. This finding is in line with alpha power in the sub-frequency bands potentially being associated with different functional roles^80^. In the lower alpha band, alpha power decreased significantly over almost the entire scalp from idea evolution to idea generation and evaluation. This finding is consistent with the figural divergent production task being associated with power decreases in the lower alpha band over almost the entire cortical sites^35^. Moreover, in line with decreases in alpha power more likely being associated with general attention demands, such as alertness and arousal^81,82^, these findings indicate heightened general attention demands during idea generation and evaluation. The less general attention demands might result from participants frequently switching their focus to connect more objects or to overcome fixation, leading to creativity during idea evolution. Similarly, alpha power desynchronized approximately 1-s before the onset of an imperative stimulus, reflecting expectancy^83^. We speculate that participants might follow their intuition to generate a solution without overcoming fixations during idea generation.

**Figure 7:**
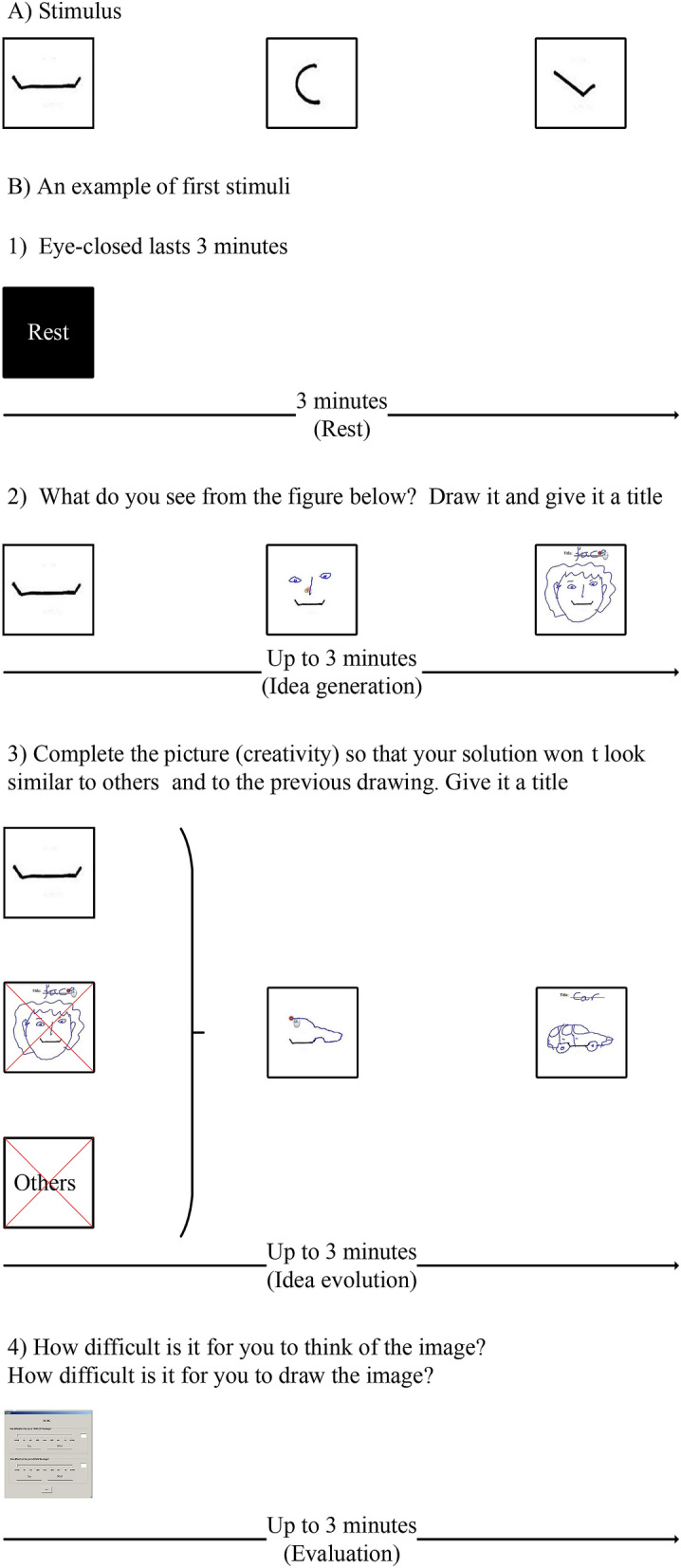
(**A**) Three different stimuli. (**B**) The first stimulus of schematic time courses of the modified TTCT-F. (1) A 3-minute rest period served as the baseline. (2) A maximal 3-minute idea generation period refers to an activation state. (3) A maximal 3-minute idea evolution period refers to an activation state. (4) A maximal 3-min evaluation period served as an activation state and a breaking fixation state.

In the upper alpha band, the power differences between areas did not exhibit a general trend for the lower alpha band in the three modes of thinking, as shown in Fig. 2. It was found that alpha power decreased more strongly in the three modes of thinking at parietal sites compared to frontal, central, and temporal sites. This finding is in agreement with findings from conventional experimental approaches in that stronger decreases in alpha power are associated with figural creative ideation at parietal and occipital sites^30,35^. In addition, this finding is consistent with that alpha power desynchronization being associated with the generation of first responses of four alternative uses^27^. Similarly, alpha power desynchronization was also observed during the verbal creative tasks fulfillment with and without overcoming self-inducted stereotypes, which lasted a few minutes^84^. Moreover, alpha power decreased significantly over temporal sites during evaluation compared to during idea evolution. Of note, differences in alpha power changes between idea generation and idea evolution were insignificant. However, this finding is inconsistent with verbal divergent thinking being associated with increases in alpha power over frontal and temporo-parietal sites (see Fink and Benedek (2014) for a comprehensive review). Recent time-course studies of divergent thinking have indicated alpha power increases at the end of the creative thinking process^23–25,27^. The insignificant differences might result from the loosely controlled creativity experiment in which idea generation might be embedded in idea evolution, which is the fundamental nature of an open-ended design process^10,12,60,85^. Alternatively, the insignificant differences might result from the adopted reference interval that was placed at the beginning of the experiment rather than the beginning of each run of the experiment. More research is needed to understand the inconsistency since little evidence is available regarding the time-course of alpha activity during the creative process in the figural domain. Indeed, previous findings have indicated that alpha power desynchronization was associated with semantic memory performance, perceptual performance, and intelligence^86–88^. Intelligence has been categorized as fluid intelligence (Gf) and crystallized intelligence (Gc), where Gf refers to the measurement of reasoning ability while Gc refers to the measurement of abilities of gaining, retaining, structuring, and conceptualizing information^89^. These findings indicated that the evaluation involved more task-specific demands such as memory and fluid intelligence since participants need to recall idea generation and idea evolution processes and evaluate their difficulty regarding thinking and drawing. Therefore, on the one hand, the loosely controlled creativity experiment appears to be valid due to consistent findings between our experiment and validated experiments at a macro level. On the other hand, the loosely controlled creativity experiment might induce some critical features of creativity, which could lead to inconsistent findings at a micro level.

**Figure 8:**
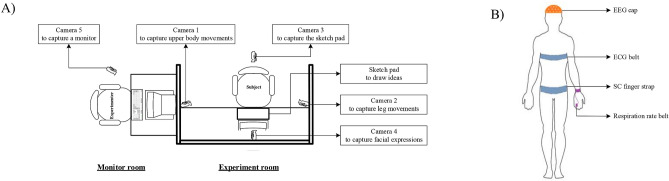
(**A**) View of the experimental configuration adapted from Nguyen and Zeng^28^ with copyright permission from Elsevier. (**B**) Configuration of the devices.

### What added value is obtained from a loosely controlled creativity experiment?

EEG microstate results revealed that multiple brain networks were activated in a different manner amid rest and the three modes of thinking. The motivation for applying EEG microstate analysis was to decompose the complex creativity activity into several primitive sub-activities through structuring the unstructured EEG data collected from the loosely controlled creativity experiment. Each primitive sub-activity was associated with a sub-phase of a creative process. The interactions between sub-activities were investigated through temporal properties of microstate classes, such as coverage and duration. Combined EEG-fMRI and EEG source localization studies have indicated that microstate classes are closely associated with the resting-state networks^50–52^. Along a similar line of thought, Benedek and Fink (2019) proposed a theoretical neurocognitive framework of creative cognition that would also need to decompose the complex cognitive capacity into different basic cognitive functions such as memory, attention, and cognitive control^90^. Benedek and Fink’s theoretical framework and the presented framework of loosely controlled creativity experiment share the same philosophy. Naturally, the new findings summarized above benefited from the loose controllability of the adopted experimental design with the support of EEG microstate analysis.

The results reported in the section ‘EEG microstate classes’ revealed six optimal microstate classes, as shown in Fig. 4. This finding supports the argument that the optimal number of microstate classes should be estimated for each individual rather than for a group by directly aggregating each individual’s EEG data^44^. Microstate classes were labelled from A to F according to the study of Custo et al. (2017), who reported seven microstate classes in the analysis of 164 subjects during rest^52^. Microstate A exhibits an asymmetric left-right orientation; microstate B exhibits a right-left orientation; microstate C exhibits an anterior–posterior orientation; microstate D exhibits a fronto-central maximum; microstate E exhibits a likely symmetric left–right orientation; and microstate F exhibits an anterior–posterior orientation. It must be noted that microstates C and F have high spatial correlations, which could lead to incorrect microstate labels between them. However, microstates C and F have negative temporal correlations compared to rest and the three modes of thinking, which could provide distinguishable information to assign their labels. This phenomenon was also observed in another study when the number of microstate classes was larger than four^52^, according to which microstate F was associated with the normative microstate C described in the study of Britz et al. (2010), while microstate C was newly generated and assigned^50^. Therefore, these findings agree with the argument that not only spatial characteristics but also temporal characteristics are necessary for assigning maps with the most appropriate microstate class^44^.

The results reported in the section ‘EEG microstate topographies’ revealed that the topographies of microstates A, B, D, E, and F were not significantly different among rest and the three modes of thinking, as shown in Table 2. These findings are in line with observations that a few resting state microstates persisted in goal-directed tasks^54^, supporting the notion that the brain might be activated in an organized manner instead of remaining inactive during rest^91,92^. Moreover, the topography of microstate C was significantly different among rest and the three modes of thinking. Microstate C has been associated with the activity in the posterior cingulate cortex (PCC), which is a central part of the default mode network (DMN)^52^. The functions of PCC appear to be associated with internally directed cognition and unconstrained rest^93^. This finding implies that activity in the DMN plays an important role during rest and the three modes of thinking. Tasks for the three modes of thinking could change the activation of DMN topology.

The results reported in the section ‘EEG microstate parameters’, revealed that the coverage and duration of microstate A increased significantly from rest to the three modes of thinking, as shown in Figs. 5 and 6. These findings are in line with the association of microstate A with visual processing since the loosely controlled creativity experiment triggers increased visual activity during idea generation and idea evolution^53,57^. However, microstate A has been associated with verbal processing at rest in a study from Britz et al.^50^. The inconsistent findings may indicate that microstate A does indeed reflect the activity in a left-posterior hub, which inhibits connections to left-hemispheric areas that are activated during verbal processing^53^. We speculate that the functional significance of EEG microstates might be distinct between goal-free and goal-directed tasks. Since little evidence is available regarding the functions of EEG microstates in task-specific conditions, more studies are needed to shed light on this issue through establishing standard databases that could record the functions of EEG microstates for each goal-free and goal-directed task.

It was found that the coverage of microstate D decreased significantly from rest to the three modes of thinking. These findings support that microstate D is associated with reflexive aspects of attention, focus switching, and reorientation, which likely occur more frequently during rest than during the goal-directed tasks^53^. Moreover, the coverage of microstate D was not significant among the three modes of thinking. Similar to this observation, the coverage of microstate D was not significantly different between fluid reasoning tasks^57^. These findings did not support another report in which microstate D was associated with the dorsal attention network, which plays an important role in spatial attention and working memory^50,94^. However, memory might be relevant for idea generation process, which is involved in recalling ideas from the memory and newly creating ideas during the tasks^95,96^. Thus, further studies are needed to better understand the functional role of microstate D and how they might be associated with memory.

Finally, the coverage of microstate C decreased significantly from idea evolution to evaluation, whereas the coverage of microstate F increased significantly from idea evolution to idea generation and evaluation. Furthermore, the duration of microstate F increased significantly from idea evolution to evaluation. As mentioned earlier, microstate C has been associated with activity in the DMN that is more active during rest. In contrast, microstate F has been primarily associated with activity in the dorsal anterior cingulate cortex (dACC), which is more active during the cognitive control tasks^50,97^. These findings support that the default mode network and cognitive control network play central roles in creativity^40,69,98^. The more active DMN indicated that idea evolution might be more associated with irrational cognitive process, such as relaxation or incubation. This irrational cognitive process might help participants overcome their fixation and redefine the open-ended problem, which is necessary for generating creative ideas. The more active control network indicated that idea generation and evaluation might be associated with rational cognitive process, such as decision-making. This rational cognitive process might help participants to determine the most appropriate solutions from several tentative solutions.

### Limitations and future directions

This study investigated the process of three modes of thinking rather than the performance of three modes of thinking in a loosely controlled creativity experiment. Further studies are needed not only to investigate brain activity during these three modes of thinking but also to assess the level of originality and efficacy of outcomes during the three modes of thinking. Even if continued exploration essentially forms creative activities, regardless of creative achievements and inconclusive outcomes, the assessment is necessary to distinguish a level of creativity and social contribution^75^. It must be noted that the assessment is a dynamic and subjective process, which depends on time, experience, and knowledge. The dynamic assessment gives rise to uncertainty that leads to creativity^75^.

In addition, the validity of loosely controlled creativity experiments is an important issue to consider. The present study addressed the issue by comparing its findings on phenomena that have been effectively studied by validated experimental research. Future studies are needed to continually verify the validity of loosely controlled experiments on creativity and other complex phenomena. These complex phenomena involve not only basic cognitive functions but also interactions among basic functions.

Finally, the presented study indicated that EEG microstate analysis is a promising tool for investigating whole-brain neuronal networks during tasks. Each whole-brain neuronal network reflects a brain function and behaves significantly different among different tasks. However, little evidence is available regarding the temporal dynamics of microstate sequences during rest^99–101^. Furthermore, to our knowledge, there is no study that aimed to uncover the temporal dynamics of microstate sequences during tasks. Understanding the temporal dynamics of microstate sequences in tasks is critical for answering Lubart’s questions: “to what extend is the creative processes recursive” and “how exactly this recursion is organized”. Therefore, additional loosely controlled creativity experiments with supporting EEG microstate analysis are needed to understand this complex phenomenon: creativity in the real-world.

### Conclusions

The present study investigated different brain responses to idea generation, idea evolution, and evaluation through a loosely controlled creativity experiment. The loosely controlled creativity experiment was designed to offer a degree of flexibility/freedom for participants to incubate the genesis of their creative ideas. The validity of the loosely controlled creativity experiment was verified through comparing its findings on the phenomena that have been effectively studied by validated experimental research. It was found that alpha power decreased significantly from rest to the three modes of thinking. These findings are in line with those from visual creativity research based on ERD/ERS and TRP. The findings of alpha power changes between three modes of thinking revealed that idea evolution required less general attention, while evaluation involved more task-specific demands, such as memory and intelligence. In addition, EEG microstate analysis revealed that microstate C was more active during idea evolution compared to during the other two modes of thinking; microstate F was less active during idea evolution compared to during the other two modes of thinking. These findings indicate that the default mode network plays a central role during idea evolution while the cognitive control network plays an important role during idea generation and evaluation. These new findings were obtained since the loosely controlled creativity experiment activates multiple brain networks to accomplish tasks involving the three modes of thinking. Taken together, the loosely controlled creativity experiment with the support of EEG microstate analysis appears to offer an effective approach to investigating real-world complex creativity activity.

## Methods

### Experimental design

The objective of this experiment was to provide a degree of freedom to simulate the flexible nature of creativity in three modes of thinking: idea generation, idea evolution, and evaluation. Not only basic creative activities but also interactions between them were simulated to form creativity. Adapted from the TTCT-F test and a work regarding sketch evolution^63^, the experiment included three conditions: idea generation, idea evolution, and evaluation^78^. A run included idea generation, idea evolution, and evaluation sequentially, which was repeated three times. Three stimuli (see Fig. 7A) were given at the beginning of idea generation for the three runs, respectively. Therefore, the experiment consisted of three runs and three conditions within each run.

During idea generation, participants were instructed to complete an incomplete sketch regarding how they had intuitively perceived the image at first sight. During idea evolution, participants were instructed to complete a sketch that was radically distinctive from what was immediately triggered by the sketch stimulus. During evaluation, participants were instructed to evaluate difficulties of thinking and drawing during idea generation and idea evolution. The duration of each condition was self-paced up to 3 min. Rest was placed at the beginning and end of the experiment, which lasted 3 min. Fig. 7B shows an example of the first stimulus of idea generation followed by idea evolution and evaluation.

### Participants and experimental procedures

Twenty-nine graduate students participated in this experiment from the Concordia Institute for Information Systems Engineering, Concordia University. Participants were offered a small gift card (a value of CAD$15) to show appreciation for their volunteering in the study after the experiment was completed. Participants were excluded from data analysis in the case of extremely noisy EEG recording. The final sample included 28 participants (4 women, 24 men) aged from 22 to 35, right-handed. All participants had normal or corrected-to-normal vision and did not report any history of medical, psychiatric disorders or treatments that could interfere any of the behavioural and neurophysiological measures. The experimenter helped participants wear EEG cap, ECG belt, respiration rate belt, and GSR finger strap. After being briefed on what to do and the impedances of all the EEG electrodes were below 10 k$$\Omega$$, the participant completed the experiment by following the experimental procedures specified in the previous subsection. During the experiment, EEG signals were recorded by a 64 channel BrainVision actiCHamp at 500 Hz. EEG was referenced to Cz, and the electrode placement was based on the international 10–10 system. Body movements, the monitor, and the subjects’ sketch pad were also captured (Fig.  8). This experimental protocol was approved by the Concordia Human Research Ethics Committee. All sections of the experiment were performed in accordance with relevant guidelines and regulations. Informed consent was obtained from all participants.

### Data analysis approach

#### Data pre-processing

EEG data were pre-processed following Harvard Automated Processing Pipeline for Electroencephalography (HAPPE)^102^. First, EEG data were filtered in the 1–50 Hz band. Second, globally bad channels of EEG data were detected and isolated using the PREP pipeline^103^. Third, eye- and muscle-generated artefacts, as well as high-amplitude artefacts of EEG data were removed using wavelet-enhanced ICA^104^. The multiple artifact rejection algorithm (MARA) was not used to address the remaining artefacts since it was trained on data collected from the international 10–20 system that was distinct from the 10–10 system. Fourth, local bad channels of EEG data were detected and isolated within each 2-s segmented epoch using FASTER^105^. Epochs of EEG data were maintained for analysis when the ratio was smaller than 0.25 between the number of bad channels and all channels. Globally bad channels and local bad channels of each 2-s segmented EEG data were interpolated from nearby channels^106^. On average, the mean number of removed bad epochs was 0.964 (SE=0.358) for rest, 0.214 (SE=0.065) for idea generation, 0.345 (SE=0.085) for idea evolution, and 0 (SE=0) for evaluation. The mean number of interpolated globally bad channels was 5.357 (SE=0.593). Finally, EEG data were re-referenced to average reference and were filtered in the 8–10 Hz, 10–12 Hz, and 1–30 Hz bands for power analysis and EEG microstate analysis, respectively. EEG data from a participant was rejected due to extreme noise in the majority of channels of their EEG recording.

#### Task-related power analysis

The power spectrum density (PSD) of EEG data in the lower (8–10 Hz) and upper (10–12 Hz) alpha frequency bands was estimated using the Welch method implemented in MNE^107^. Each time window consists of 1000 sample points while 500 sample points were overlapping between two neighbouring time windows. Alpha power at each electrode in each run of conditions was estimated using the composite Simpson’s rule. Alpha power changes between conditions were quantified using the following Eq. (1):

1 $$\begin{aligned}&TRP_{ki}= Mean\left( log\left( Pow_i,activation\right) _{kj}-log\left( Pow_i,reference\right) _{kj}\right) , \nonumber \\&\qquad i\in [1, n\_chs], j \in [1, n\_runs], k\in [1, n\_conditions], \end{aligned}$$

where *k* is the index of conditions ranging from one to three corresponding to idea generation, idea evolution, and evaluation, respectively; *j* is the index of runs in each condition ranging from 1 to 3; *i* is the index of EEG channels ranging from 1 to 63.

Compared to the existing studies, TRP results at 63 electrodes were aggregated into the five cortical areas at left and right hemispheres^27^. In the left hemisphere, the frontal area includes the following electrodes: Fp1, AF3, AF7, F1, F3, F5, F7, FC1, and FC3. The central area includes the following electrodes: FC5, C1, C3, and C5. The temporal area includes the following electrodes: FT7, T7, TP7, CP5, and P5. The parietal area includes the following electrodes: CP1, CP3, P1, and P3. The occipital area includes the following electrodes: PO3, PO7, P7, and O1. In the right hemisphere, the frontal area includes the following electrodes: Fp2, AF4, AF8, F2, F4, F6, F8, FC2, and FC4. The central area includes the following electrodes: FC4, C2, C4, and C6. The temporal area includes the following electrodes: FT8, T8, TP8, CP6, and P6. The parietal area includes the following electrodes: CP2, CP4, P2, and P4. The occipital area includes the following electrodes: PO4, PO8, P8, and O2. In addition, the midline electrodes were not included in subsequent statistical analysis.

#### Microstate analysis

Identification of microstate classes was accomplished by a modified version of the k-means^45^. For each participant, in each condition, and in each run, the global field power (GFP) that is the standard deviation of the potential of a map was calculated based on Eq. (2). The map topographies corresponding to peaks of GFP were submitted to the modified k-means algorithm due to their having the highest topographic stability^46^. The number of k was predefined from 1 to 10. For each k, the modified k-means was repeated 100 times. The representative map topographies were determined by minimizing the cost function based on Eq. (3). The optimal number of representative map topographies was determined by the cross-validation criterion based on Eq. (4). Therefore, individual classes of representative map topographies were determined for each participant, for each condition, and for each run:

2 $$\begin{aligned} GFP= \,& {} \sqrt{\frac{\sum _{i=i}^{N_S} (u_i-{\overline{u}})^2 }{N_S}}, \end{aligned}$$

3 $$\begin{aligned} F= \,& {} \frac{1}{N_T(N_S-1)}\sum _{t=1}^{N_T}||V_t - \sum _{k=1}^{N_K}a_{kt}\Gamma _k ||^2, \end{aligned}$$

4 $$\begin{aligned} CV=\, & {} \frac{\sum _{t=1}^{N_T} (V_t'\cdot V_t - (V_t'\cdot \Gamma _k)^2)}{N_T(N_S-1)}\cdot (\frac{N_S-1}{N_S-1-N_K})^2, \end{aligned}$$

where $u_i$ is the electric potential of the map *u* at the electrode *i*, ${\overline{u}}$ is the average electric potential of all electrodes of the map *u* and $N_S$ is the number of electrodes of the map *u*. $N_T$ is the duration of each run. $V_t$ is a $N_S \times 1$ vector consisting of the electric potential at time instant *t*. $N_K$ is the number of representative map topographies. $\Gamma _k$, which is a normalized $N_S \times 1$ vector, represents the k-th representative map topographies. $a_{kt}$ is the intensity of the k-th representative map topographies at the time instant t.

To conduct group-level analysis, individual classes of map topographies were used to compute the mean classes of map topographies. The mean classes were determined through maximizing spatial correlations with a full permutation procedure between individual classes and mean classes^108^. Specifically, each individual class of map topographies must be assigned into different mean classes in that they were labelled the same. The polarities of spatial correlation were ignored since oscillations of the same neural generator may result in the inversion of scalp potential filed^44^. The same procedure was applied to compute mean classes across runs, followed by computing mean classes across participants and computing mean classes across conditions.

As a result, global microstate classes were determined to fit backward to the original map topographies at peaks of GFP based on maximal spatial correlation. Successive original map topographies corresponding to peaks of GFP with the same labelled class were identified to one microstate class. The first microstate class of each 2-s epoch was removed because it is not known when it started. The last microstate class of each 2-s epoch was removed since it is not known when it ended.

The coverage and duration of microstate classes were calculated to explore relevant properties of microstate class sequences. The coverage is the percentage of recording time covered by each microstate, while the duration is the average time of each stable microstate. Microstate parameters were computed for each run, each condition, each participant, and each microstate class through averaging the parameters within all 2-s epochs.

#### Statistical analysis

First, task completion time was analysed by repeated measures ANOVA considering RUN (the first run, second, and third runs) of each condition as a within factor. In addition, task completion time was analysed by repeated measures ANOVA considering CONDITION (idea generation, idea evolution, and evaluation) as a within factor. A paired t-test was used to determine differences in task completion time between runs of a condition, as well as among conditions.

Second, alpha power changes were analysed by a $3 \times 5 \times 2$ repeated measures ANOVA with three within factors CONDITION (idea generation, idea evolution, and evaluation), AREA (frontal, central, temporal, parietal, and occipital in each hemisphere), HEMISPHERE (left and right). Post hoc comparisons were performed with Bonferroni correction.

Third, microstate class topographies were analysed using Topographic ANOVA (TANOVA) through a randomization test^109,110^. Specifically, a TANOVA with two factors was conducted considering CONDITION and MICROSTATE CLASS as independent variables and topography as a dependent variable, which is followed by a class-wise TANOVA with one factor considering CONDITION as an independent variable and topography as a dependent variable. Post hoc comparisons were performed using paired TANOVAs.

Fourth, microstate class parameters (mean duration and mean coverage) were analysed by a $4 \times K$ repeated measures ANOVA with two within factors CONDITION (rest, idea generation, idea evolution, and evaluation) and CLASS (*K*). *K* is the number of optimal microstate classes. Post hoc comparisons were performed using the paired t-test.

During the statistical analysis above, post hoc comparisons were performed to reveal additional fine-grained results. Greenhouse-Geisser correction was applied in case of violations of the sphericity assumption.

## Data Availability

The datasets generated and analysed in the current study are available in the G-Node repository, https://gin.g-node.org/Design-Lab/EEG-signals-respond-differently-to-three-modes-of-thinking-in-a-loosely-controlled-experiment.
